# New Analytical Strategies for Quality Control and Classification of Apple Juices Using Digital Image Processing (DIP) Combined with Machine Learning (ML)

**DOI:** 10.1021/acsomega.5c08212

**Published:** 2025-12-17

**Authors:** Suelem Kaczala, Vanderlei Aparecido de Lima, Maria Lurdes Felsner

**Affiliations:** † Department of Chemistry, 307046 State University of Midwestern at Paraná (UNICENTRO), Vila Carli, Guarapuava, Paraná 85040-080, Brazil; ‡ Department of Chemistry, 74354 Federal University of Technology − Paraná (UTFPR), Pato Branco, Paraná 85503-390, Brazil; § Department of Chemistry, State University of Londrina (UEL), Londrina, Paraná 86057-970, Brazil

## Abstract

Apple juice is widely consumed for its pleasant sensory attributes and nutritional value. However, due to its high commercial value, this beverage, particularly whole juice, is susceptible to fraud and adulteration. This underscores the need for rapid, noninvasive analytical strategies to ensure product authenticity and quality. This study reports, for the first time, the application of smartphone-based image analysis combined with machine learning as a low-cost and nondestructive approach for both classifying apple juice types and predicting the actual juice content in apple-based beverages. Images of nine whole juice (WJ), four reconstituted juice (RE), and five nectar (NE) samples were analyzed to develop models capable of discriminating beverage categories and estimating apple juice concentration. Classification models generated using *k*-nearest neighbors (*k*NN) and extreme gradient boosting (XGBoost) algorithms achieved good global accuracies of 95.9% (99.1% and 100.0% in training and 95.9% in testing), with cross-validation accuracies of 98.7% and 95.5%, respectively. Predictive models constructed from calibration curves (5–100% apple juice) combined with commercial samples yielded good estimates for coefficients of determination (*R* ^2^ = 93.1–93.5% in testing, 97.7–96.2% in training, and 91.9–92.4% in cross-validation) and root-mean-square errors (RMSE = 8.0–8.2%) for models generated by XGBoost and CatBoost, respectively. These findings demonstrate the scientific novelty and practical feasibility of integrating smartphone imaging with machine learning for the quantitative analysis of apple juice. The proposed approach represents a rapid, accurate, and cost-effective alternative for industrial quality control and regulatory inspection.

## Graphical abstract

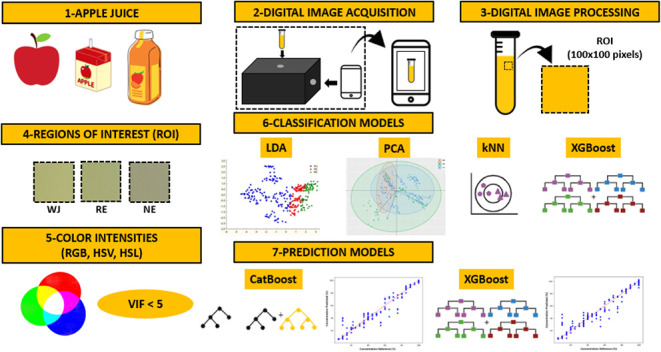

## 1 Introduction

It is well established in the literature that fruit consumption plays a crucial role in maintaining a healthy diet and preventing the development of various diseases. Fruits are rich sources of nutrients with recognized health-promoting properties, including vitamins, fibers, bioactive compounds, and minerals.^1,2^ Among fruits, apples stand out due to their high nutritional value, which can be transferred to some of their derived products, such as whole juices, depending on the technological processing applied.^3^ In terms of consumer preference, apple juice ranks as one of the most consumed fruit juice globally, appreciated for its authentic and distinct flavor. It is recognized for its health benefits attributed mostly to its richness in phenolic compounds. These compounds contribute to its demonstrated biological effects including antiatherosclerotic, anti-inflammatory, and neuroprotective effects.^4^

In recent years, lifestyle changes have significantly influenced eating habits across populations, leading to a reduction in meal preparation using fresh ingredients and, consequently, in the consumption of fresh fruits. This trend has been particularly pronounced among low- and middle-income groups, who increasingly favor ready-to-eat or ready-to-drink products, such as commercial fruit-based beverages.^5^ Conversely, an emerging group of consumers has shown heightened concern regarding the quality and nutritional value of the beverages they consume, as well as the sustainability of production processes.^1^ These contrasting perceptions of fruit juice quality have driven the food industry to diversify its portfolio, offering fruit-based beverages with distinct nutritional and compositional characteristics.^4^ Consequently, several types of fruit-based beverages are available on the market, including whole juice, reconstituted juice, and nectar, between others.

Whole juice is obtained directly from the fruit and does not contain added sugars or other additives. It is typically labeled as “100% juice,” although, in industrial practice, limited dilution may be performed to comply with the specific regulatory standards established for each fruit type.^6,7^ Reconstituted juice is produced by rehydrating concentrated juice with water to achieve a soluble solids content equivalent to that of whole juice. For apple reconstituted juice, the minimum soluble solids content (°Brix) required is 11.5 according to the Codex Alimentarius (2005)^6^ and 11.2 according to the European Commission (2009).^7^ Nectars, on the other hand, are nonfermented beverages that may contain between 25% and 50% juice, depending on the fruit used, and may include added sugars and other authorized ingredients.^6^ To be classified as apple nectar, the beverage must contain at least 50% apple juice.^6^ However, under Brazilian legislation, a minimum apple juice content of 20% is required for this category of fruit-based beverage.^8^

Due to their superior nutritional and organoleptic attributes compared with reconstituted juices and nectars, whole juices have attained greater commercial value, consequently becoming more vulnerable to fraudulent practices and adulteration.^9–11^ Among fruit juices, apple and orange juices are among the most frequently subjected to such unscrupulous practices.^12^ The most common form of adulteration involves the addition of water and sugars, which can typically be identified by physicochemical analyses due to alterations in the composition and organoleptic characteristics of the beverage.^9,10,13^ However, recent studies have reported that fraud and adulteration in fruit juices have become increasingly sophisticated, involving subtle compositional modifications that are not easily detected by spectrometric, chromatographic, or physicochemical analyses.^9,10,13,14,16^

In this context, novel strategies aimed at curbing fraud and adulteration and ensuring consumer safety have been reported. These include the classification of apple juices according to fruit varieties, processing conditions, and geographical origin.^10,12,14–19^ However, to date, the classification of apple-based beverages into whole, reconstituted, or nectar categories, as well as the determination of the fruit juice content in these beverages, has not been reported.

The application of image analysis for beverage quality control has gained considerable attention in recent years. This approach has been employed for a range of beverages, including soft drinks,^20^ milk,^21^ alcoholic beverages,^22^ and fruit juices.^23^ Methodologies incorporating digital image processing (DIP) offer significant environmental benefits and several advantages over spectrometric and chromatographic methods, including greater analytical speed, minimal sample requirements, simple instrumentation, low cost, and reduced waste generation.^24^ However, a limitation of image analysis is its inability to directly detect subtle changes in sample composition. This limitation can be addressed by integrating image-derived data with chemometric techniques such as machine learning (ML), thereby enhancing discriminatory power.^9,25,26^

The integration of DIP and ML has enabled the development of rapid, cost-effective, and reliable classification and prediction strategies for various food products, including beverages,^20^ brown sugar,^25^ and pollen.^26^ These approaches have proven effective in addressing complex analytical and authentication challenges. However, to the best of the authors’ knowledge, no strategy has yet been proposed to classify apple-based beverages in their various commercial forms or to predict the apple juice content added to each beverage category using the DIP–ML combination. Therefore, this study aims to develop and evaluate ML models using DIP data to (i) classify apple juice samples as whole (WJ), reconstituted (RE), and nectar (NE), and (ii) predict the concentration of apple juice in different product types.

## 2 Materials and Methods

### 2.1 Sampling

Eighteen apple juice samples collected between 2022 and 2023 were purchased from supermarkets and other commercial establishments to represent a wide variety of brands, processing methods, and formulations available in the Brazilian market, ensuring that no brand was repeated within each category. Seventeen samples were obtained from different manufacturers, while two originated from the same company, one nectar and one whole juice.

The samples included whole juices, reconstituted juices, and nectars, available in both clarified and cloudy forms. Their compositions, as declared on the labels, and the assigned laboratory codes are provided in (Table S1 Supporting Information). Samples were coded alphanumerically according to beverage type (whole juice, WJ; nectars, NE; and reconstituted juices, RE), processing form (clear, C; cloudy, WC), and order of arrival at the laboratory. They were stored under refrigeration (6–7 °C) until the analyses were carried out.

Classification as nectar, reconstituted juice, or whole juice was based on label information (Table S1, Supporting Information) and confirmed via physicochemical analyses of reducing sugars and elemental composition (Na and K), following Kaczala, Lima, and Felsner (2025).^27^ Apple juice concentrations ranged from 10% to 100%, depending on processing and beverage category (Table S1). According to Brazilian legislation, whole juices retain their natural concentration (100%), reconstituted juices are restored to original concentration by adding water, and nectars contain a minimal of the 20% juice.^6,8^ The limited number of samples reflects the predominant use of apple juice in mixed fruit beverages in Brazil, which reduces added sugar and balances sweetness and acidity, as well as the seasonal availability of apple juice.

### 2.2 Digital Image Acquisition and Processing

A rectangular cardboard box measuring 32 cm in length, 26 cm in width, and 12 cm in height was modified to serve as a closed chamber for image acquisition. It featured a 1.5 cm opening at the top to accommodate an 8 mL capped test tube containing the apple juice sample, and a rectangular side opening (2 cm × 1.5 cm) to allow the placement of a smartphone camera for image capture, as illustrated in (Figure S1 Supporting Information). The interior of the box was lined with white paper to minimize shadows and reflections on the test tube.

A qualitative study was carried out to optimize the lighting conditions inside the image capture chamber. Two LED flashlights (A, 1,600 lm, and B, 800 lm) were positioned at different locations within the box relative to the test tube containing the apple juice sample. The diagonal arrangement, with LED flashlight A placed in front of the test tube and LED flashlight B positioned behind it, provided the lowest incidence of shadows and reflection points from the light source on the glass surface. Therefore, this configuration of LED flashlights (A and B) was considered optimal and adopted in subsequent experiments (Figure S1).

The distance between the camera and the glass bottle containing the sample was set to 12 cm, following recommendations from the literature.^28,29^ This distance was considered optimal as it avoided the need for zooming or manual focus adjustment of the smartphone camera prior to image acquisition. The images were captured using the 64-megapixel (MP) rear camera of a Samsung A52 smartphone, with a resolution of 2400 × 1080 pixels, and without the use of any external flash source as illustrated in Figure S1. One limitation of the optimization study conducted in this work is that real apple juice samples were used instead of reference samples to determine the optimal conditions for image acquisition. In addition, the influence of colorant presence in apple juice on the intensity of chromatic variables was not evaluated.

To build the models for classifying apple juices, three images were acquired for each of the 18 samples, totaling 54 images (Figure 1). Each image was digitally cropped using the free license software Gimp v.2.10.32 ^30^ into six regions of interest (ROI) with dimensions of 100 × 100 pixels (Figure S2), totaling 324 croppings. For the creation of classification models, three classes were adopted: whole juice (WJ), reconstituted juice (RE), and nectars (NE).

**Figure 1:**
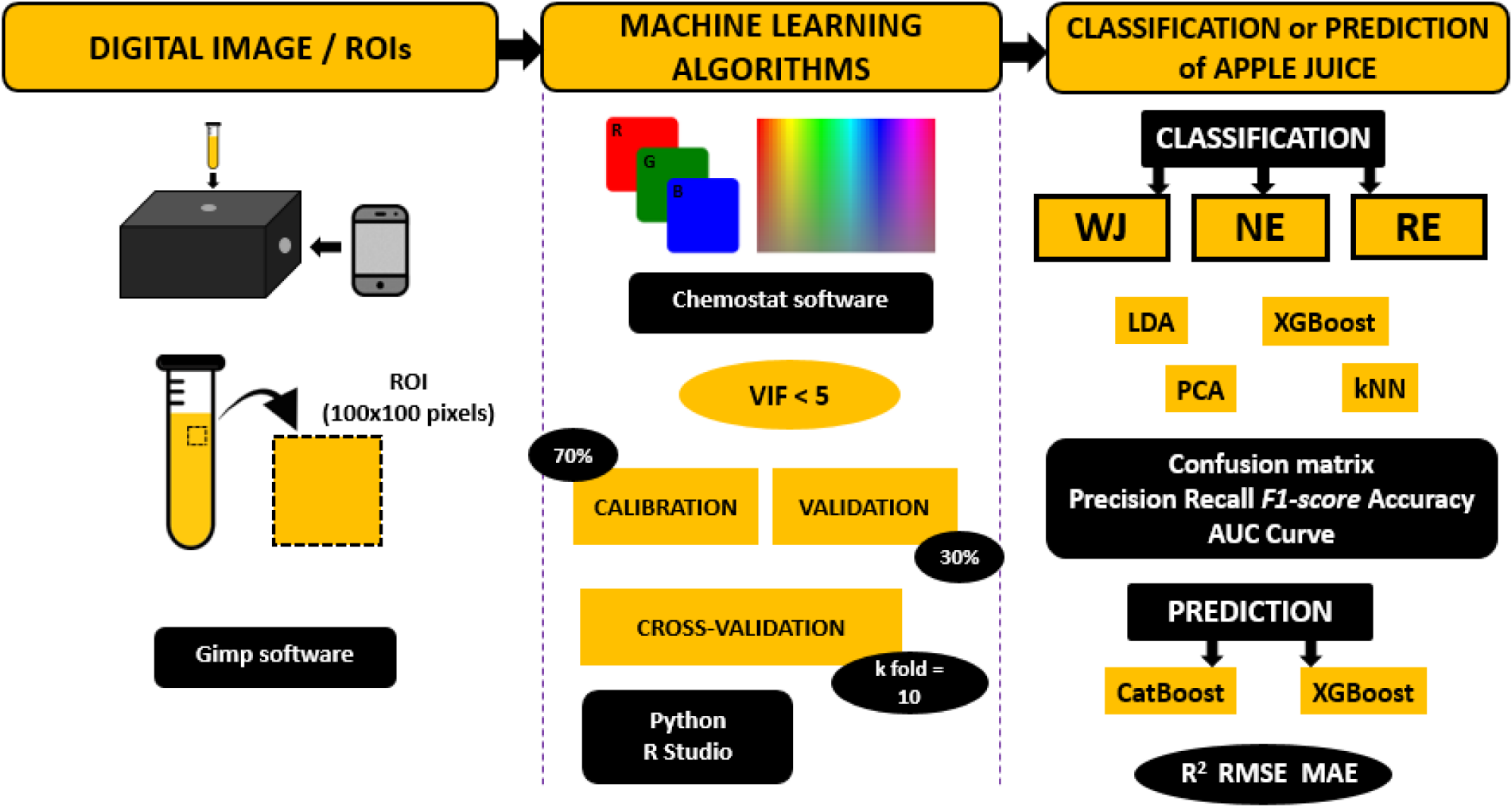
Illustration of the approaches used to classify apple juice as whole juice (WJ), nectar (NE), and reconstituted juice (RE) categories and to predict apple juice concentration (%) in apple beverages using machine learning algorithms.

In order to construct a model for predicting apple juice concentrations (%) in the various presentations of this beverage (WJ, RE, and NE), a calibration curve was generated using a composite sample of apple juice. This composite sample was prepared by mixing 100 mL of five randomly selected commercial whole apple juice samples (WJ1, WJ3, WJ7, WJ8, and WJ9), resulting in a total volume of 500 mL. As shown in Table S1, these five samples originated from distinct manufacturers and represented different chemical compositions and technological processing types. Among them, three had undergone filtration/clarification processes, while two were unfiltered/unclarified. According to the information provided by the manufacturers (Table S1), the selected samples also contained different ingredients listed on their labels, which could mimic the compositional variations typically observed in other juice categories (RE and NE).

A calibration curve was constructed in the concentration range of 5 to 100% (v/v) by varying the proportions of the composite sample and distilled water (Table S2). Each calibration solution received a mass of sodium metabisulfite corresponding to a final concentration of 50 ppm. The addition of this compound was intended to better simulate the composition of commercial apple juices.

In the juice industry, additives such as sodium metabisulfite, ascorbic acid, and citric acid are widely used as color-stabilizing agents in products including apple juice. These additives inhibit the activity of polyphenol oxidases (PPO), the enzymes responsible for enzymatic browning, and prevent the formation of quinones that, upon polymerization, produce brown pigments and alter juice color during storage. Because these compounds are often incorporated into nectars and reconstituted juices, but are not always present in whole juices, it was important to include one or more of them in the calibration curve solutions prepared from composite sample. This approach ensured that, when such additives were present in real samples, the calibration solutions reproduced the intrinsic color variability associated with the specific technological processes used in each beverage category, thereby minimizing uncertainties in the model’s predictive accuracy.

Subsequently, a volume of 4.0 mL from each calibration curve solution (Table S2) was transferred to a test tube, which was then inserted into the image acquisition chamber (Figure S1). Three images were obtained for each standard solution (Table S2), resulting in 60 images. Each image was divided into six regions of interest (ROIs), with dimensions of 100 × 100 pixels, resulting in 360 ROIs (Figure S2).

The ROIs for classification and prediction modeling were imported into the free-license software Chemostat v.2 ^31^ to separate and quantify the color intensities of the RGB channels and the HSV, HSL, and HSI color spaces. The RGB channels generate color index histograms of the apple juice samples (Figure 1).

### 2.3 Modeling by Machine Learning Algorithms

To identify the most critical variables for classification and prediction modeling and to mitigate the influence of multicollinearity in the data, a Variance Inflation Factor (VIF) analysis^32,33^ was performed in the R v.4.2.1 program.^34^ Consequently, all variables exhibiting VIF values >5 were excluded from the dataset (Figure 1).

The dataset, comprising 324 ROIs, was divided into two subsets. Seventy percent (*n* = 226) of the data were used to construct the training model, while the remaining 30% (*n* = 98) were reserved for testing. To ensure statistical rigor, all samples were assigned to the training and test sets using a randomized procedure. After data preprocessing, the predictors and target variables were split using the train_test_split function from the *scikit-learn* library: X_train, X_test, y_train, y_test = train_test_split (predictors_scaled, target, test_size = 0.3, random_state = 0). This function performs a fully randomized allocation of samples while maintaining reproducibility through the fixed random_state parameter. Consequently, multiple ROIs extracted from the same physical bottle were not manually grouped; instead, they were randomly assigned to either the training or test partition.

Subsequently, the two data subsets comprising the color intensities of the R and G channels, previously selected based on the LDA and VIF analyses, were subjected to classification modeling using the *k*NN and XGBoost algorithms (Figure 1).

Two supervised machine learning algorithms, Extreme Gradient Boosting (XGBoost) and *k*-Nearest Neighbors (*k*NN), were applied for multiclass classification using Python libraries (XGBoost and scikit-learn). Model optimization for both algorithms was conducted through grid search (GridSearchCV) combined with 5-fold stratified cross-validation (StratifiedKFold) to maintain balanced class proportions. The F1-macro score was adopted as the optimization criterion to account for class imbalance. For XGBoost, the model was configured with a softmax objective function (multi:softprob) and evaluated using the multiclass logarithmic loss (mlogloss) metric, exploring combinations of maximum tree depth (*n* = 3 and 6), learning rate (0.05, 0.1), number of estimators (200, 500), subsample ratio (0.8, 1.0), and column sampling rate (colsample_bytree = 0.8, 1.0). For *k*NN, the search included neighborhood sizes (n_neighbors = 3–13), distance weighting strategies (uniform, distance), and distance metrics (Euclidean, Manhattan, Minkowski) with corresponding Minkowski power parameters (*p* = 1 and 2). The best models, selected according to the highest cross-validated *F1-macro scores*, were retrained on the full training set and evaluated on an independent test set using overall accuracy, *F1-macro score*, classification metrics, and confusion matrix visualization.

Cross-validation was applied to the dataset using the *k*-fold method with *k* = 10 for each algorithm. To obtain a robust estimate of generalization, classification models were evaluated using a train–test split and 10-fold cross-validation. The performance of the generated classification models was evaluated through confusion matrices, AUC curves (Figures S3 and S4) and the figures of merit (accuracy, precision, recall, and *F1-score*), as described by Alves et al.^25^

In order to build a predictive model, the dataset (*n* = 684), considering the data extracted from the images of the analytical curve (*n* = 360), the apple juice samples (*n* = 324) (Figure 1) and the variables identified with VIF values <5 (G, B, and H), was divided into two subsets: one for training purposes (comprising 70% of the data, *n* = 479) and the other for testing purposes (comprising 30% of the data, *n* = 205). Additionally, the five whole-juice samples used for constructing the calibration curve were not excluded from the test set. Because the purpose of our modeling approach was to evaluate predictive performance under realistic and heterogeneous conditions, these samples were randomly allocated in the same manner as all other samples. This procedure ensures that model validation reflects natural variation across brands and batches.

The two subsets were then subjected to predictive modeling using the CatBoost and XGBoost algorithms. Again, to obtain a robust estimate of generalization, prediction models were evaluated using a train–test split and 10-fold cross-validation. The prediction models were also evaluated using three key metrics: coefficient of determination (*R* ^2^), root-mean-square error (RMSE), and mean absolute error (MAE). All algorithms were executed in Python v.3.8.^35^

### 2.4 Multivariate Analysis

To identify grouping patterns associated with the types of apple juice (WJ, NE, and RE), a principal component analysis (PCA) was performed on the variables previously selected (H, L, R, and G) based on the VIF analysis. All data were mean-centered, and the correlation matrix was chosen. A supervisioned technique, the Linear Discriminant Analysis (LDA) was also carried out using the intensities of R and G channels of RGB space color for classification supervisioned of apple beverages in WJ, RE, and NE. These analyses were conducted using the open-source R software.^34^

## 3 Results and Discussion

### 3.1 Color Characteristics of Apple Whole Juices, Reconstituted Juices, and Nectars

Apple juices exhibited color variations ranging from light yellow and orange to golden hues, which are closely related to their chemical composition and the technological processing applied. Commercially, apple juices are available in different forms, including whole juices (WJ), reconstituted juices (RE), and nectars (NE), which may be either clarified or cloudy. Cloudy juices, in particular, typically displayed a golden hue resulting from interactions between proteins and colloidal particles suspended in the juice.^36^ This behavior was observed in this study for the cloudy WJ and RE samples (Figure S2). Conversely, clarified juices tended to be lighter in color (light yellow to orange) due to the removal of components responsible for the characteristic coloration of apple juice (Table S1, Figure S2).

Technological factors involved in producing WJ, RE, and NE further influenced their final color, as these beverages are processed under different conditions. NE and RE are generally prepared by diluting concentrated, filtered or unfiltered apple juice with water, followed by the addition of other compounds. Enzymatic and nonenzymatic browning reactions during pasteurization and concentration steps can lead to the formation of colored pigments, which also affect the final color of the beverage. Consequently, the final color of apple juice results from a complex interplay of factors, including the action of polyphenol oxidase (PPO) on substrates such as chlorogenic acid present in the must, the oxidation of this acid to quinones, the presence of stabilizing agents, and the interactions between colloidal particles and proteins, as well as the technological processes applied to clarified and cloudy juices.^36^

To evaluate the clustering patterns observed among WJ, NE, and RE, which differ in processing and composition, a Principal Component Analysis (PCA) was conducted using the intensities of the R, G, H, and L variables extracted from the images and previously selected through variance inflation factor (VIF) analysis. The first two principal components explained 91.5% of the total variance, with PC1 (52.3%) associated primarily with R and G variables, and PC2 (39.2%) positively correlated with H and L variables (Figure 2). Three distinct clusters were identified: the first included WJ and RE samples with higher R and G values; the second cluster comprised NE and RE samples with elevated H and L values; the third cluster contained samples from all three categories. Under Brazilian legislation, nectars are required to contain a minimal of the 20% apple juice.^8^ In this study, the most nectar samples reported declared apple juice concentrations >35% (Table S1). This may explain the overlap between the three clusters on the PCA. A greater dispersion of RE samples across PCA quadrants (Figure 2) indicated that manufacturers adopt different technological processes in their formulation, resulting in behavior similar to both NE and WJ samples. Given the higher commercial value of WJ, achieving a clear distinction between categories is important to prevent fraud and adulteration.

**Figure 2:**
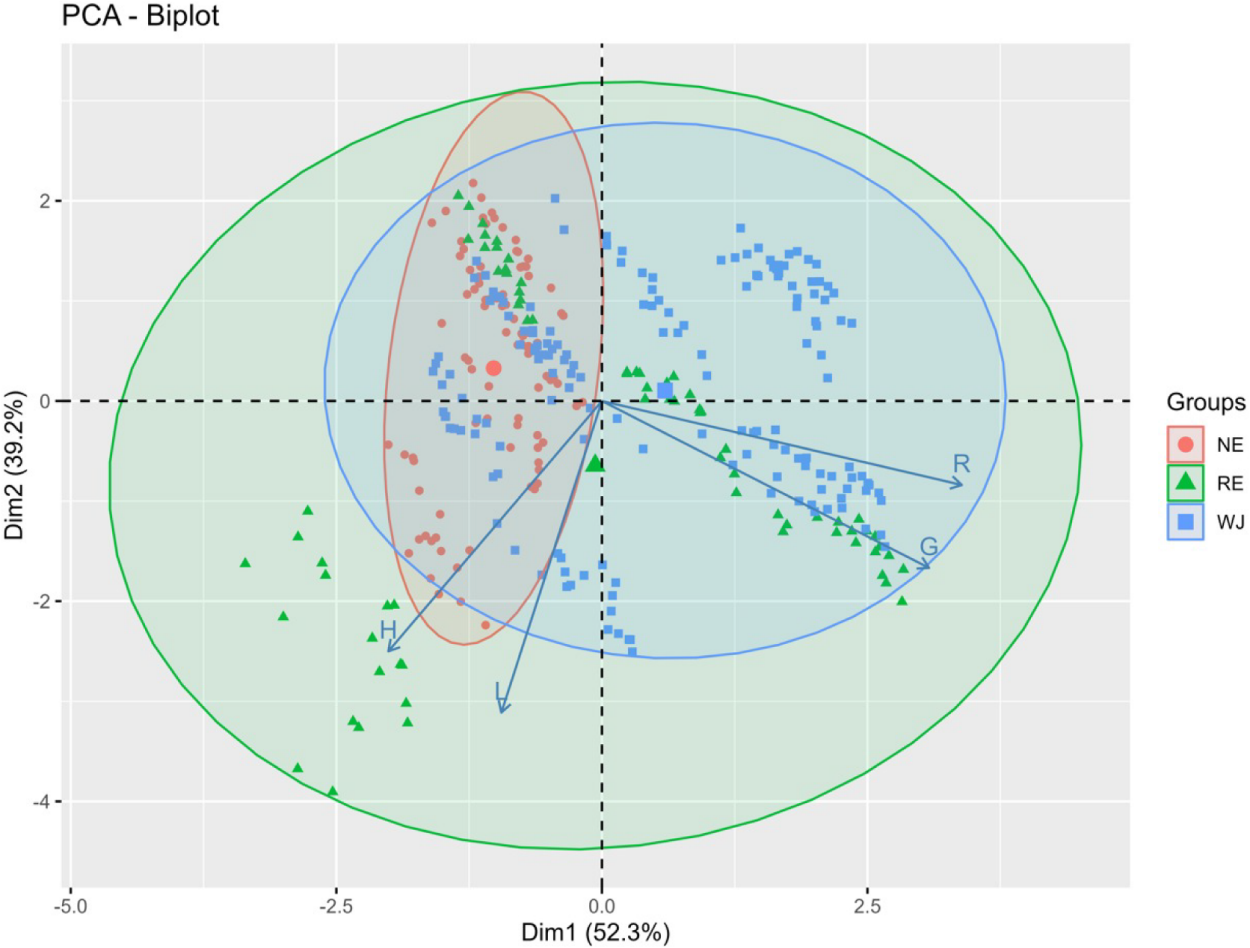
Projection of loadings and scores on the first two principal components, PC1 and PC2, for grouping of nectar (NE), reconstituted juice (RE), and whole juice (WJ) samples.

To classify the apple juice samples into different categories (WJ, RE, and NE), initially, a linear discriminant analysis (LDA) was performed using only the R and G variables, which exhibited the greatest variability among the evaluated classes, as illustrated in PCA plot (Figure 2). The first canonical function exhibited a high eigenvalue (2.7955) and a strong canonical correlation (*R* = 0.8582), indicating that approximately 73.7% (*R* ^2^ = 0.737) of the variance in the discriminant scores was explained by differences among the groups (Figure 3). The corresponding Wilks’ Lambda value (0.2591) was relatively low, confirming substantial differences among the categories along this function. The associated Chi-square test (χ^2^ = 432.85, d*f* = 4, *p* < 0.001) further demonstrated that this discriminant function significantly contributes to class separation.

**Figure 3:**
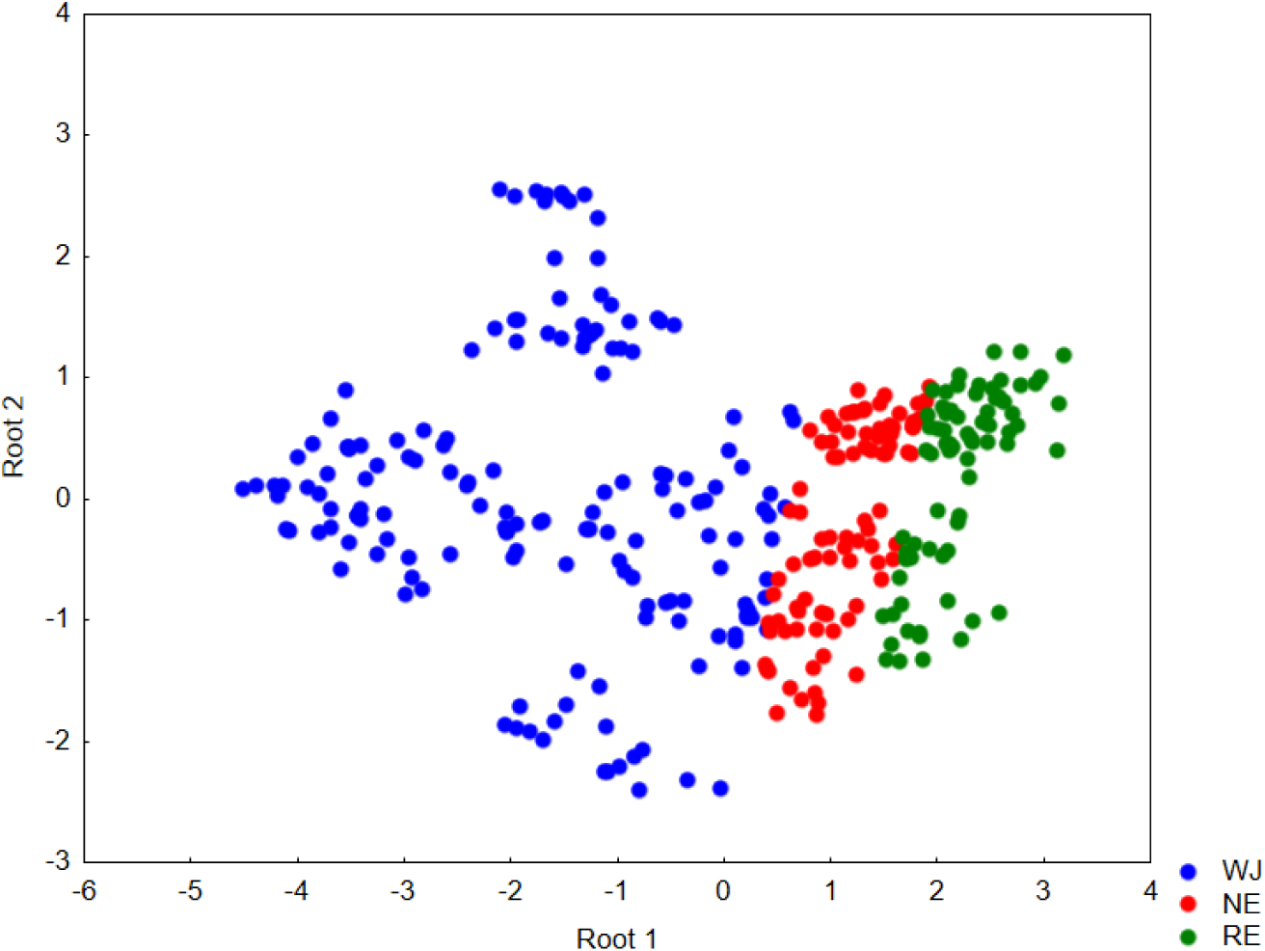
Two-dimensional plot of the intensity of scores of the R and B variables extracted from the images of apple whole juice (WJ), reconstituted juice (RE), and nectar (NE) samples based on the two discriminant functions.

In contrast, the second discriminant function presented a very low eigenvalue (0.0169) and a weak canonical correlation (*R* = 0.1287, *R* ^2^ = 0.017), explaining only about 1.6% of the total variance. Although its Chi-square value (χ^2^ = 5.36, d*f* = 1, *p* = 0.021) was statistically significant, the Wilks’ Lambda (0.9834) indicated a negligible contribution to the overall discrimination (Figure 3). Taken together, these findings suggest that the first discriminant function accounts for nearly all of the meaningful group separation (≈99.4% of the total explained variance), whereas the second function provides minimal additional discriminatory power. Therefore, group differentiation in this dataset is essentially captured by the first canonical axis.

The discriminant classification results showed an overall correct assignment rate of 83.3% (270 out of 324 samples), confirming the strong group separation previously indicated by the canonical discriminant analysis (Figure 3). Classification accuracy varied across the three categories: WJ was correctly classified in 84.6% of cases (137 out of 162), with all misclassified samples assigned to the NE group; NE achieved the highest accuracy (88.9%), with 10 samples misclassified as RE and none as WJ; and RE exhibited the lowest accuracy (73.6%), with 19 samples incorrectly classified as NE.

Regarding precision, all samples predicted as WJ were indeed WJ (100% precision). In contrast, RE showed intermediate precision (84.1%), whereas NE exhibited lower precision (64.5%) due to receiving most of the misclassified samples from both WJ and RE. This asymmetric error pattern indicates that misclassifications were concentrated toward the NE group, suggesting that NE occupies an intermediate position between WJ and RE in the canonical space (Figure 3). Consequently, WJ appears well separated from the other groups, while NE partially overlaps with both extremes, consistent with the structure of the first canonical function, which accounted for nearly all the explained variance.

The analysis employed prior probabilities of 0.50 (WJ), 0.2778 (NE), and 0.2222 (RE). Despite the higher prior for WJ, the model did not overassign samples to this group, demonstrating that class discrimination was primarily driven by genuine differences in the predictor variables rather than by prior bias. Although the overall performance was satisfactory, the lower precision observed for NE suggests partial overlap among classes. Future analyses could explore additional variables, nonlinear discriminant functions, or cost-sensitive classification rules to further reduce classification ambiguities.

Building upon these findings, machine learning (ML) algorithms were subsequently employed to enhance classification accuracy and prediction robustness. Unlike LDA, which assumes linear separability among classes, ML approaches such as *k*NN, XGBoost, and CatBoost can capture complex, nonlinear relationships between image-derived color features and beverage categories. The application of these advanced algorithms aimed to improve the discrimination between WJ, RE, and NE, and to accurately predict the apple juice content in each beverage type, thereby extending the analytical capability of smartphone-based image analysis toward a fully automated and scalable authenticity assessment tool.

### 3.2 Classification Modeling of Apple Juice

To evaluate the potential of machine learning algorithms for classification tasks, two algorithms, *k*NN and XGBoost, were applied to the R and G intensities extracted from digital images with the objective of classifying apple juice samples into WJ, RE, and NE categories. The choice of these algorithms for modeling was embased in following reasons. *k*-Nearest Neighbor (*k*NN) is widely used for classification and prediction, particularly in the food and beverage industry.^37,38^ It is a supervised algorithm that assigns a class to an unknown sample based on the majority class among its nearest neighbors, as determined by the Euclidean distance.^39,40^ This method is particularly suitable for small datasets, provided that class sizes remain balanced during training. Extreme Gradient Boosting (XGBoost) is a decision tree-based ensemble algorithm that improves predictive accuracy by sequentially correcting classification errors from previous trees.^41^ This approach produces models with high accuracy and precision and minimal residuals when classifying unknown samples.^42^ It is recognized for its high training efficiency, strong predictive performance, capability to optimize both data and model parameters, and reduced overfitting tendency.^41,43^

The *k*NN-based model exhibited excellent performance in classifying all juice categories (WJ, RE, and NE) (Figure 4). The highest classification accuracy was observed for the RE class, with 55 out of 55 instances correctly classified in the training stage and 17 out of 17 during external validation. For the WJ class, 107 out of 108 instances were correctly assigned during training and 52 out of 54 during external validation. In both stages, some misclassifications occurred between WJ and NE samples. For the NE class, the algorithm also achieved outstanding performance, correctly classifying 62 out of 63 instances in the training stage and 25 out of 27 in the testing stage. Similarly, some misclassifications were observed between NE and RE samples.

**Figure 4:**
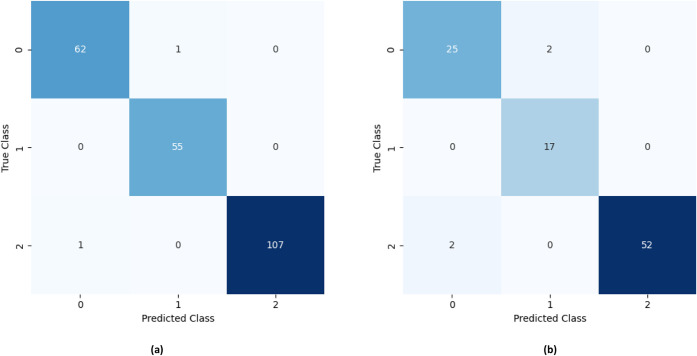
Confusion matrix for the classification model developed by the kNN algorithm, considering the (a) training and (b) testing stages. Code for classes: NE = 0; RE = 1; WJ = 2.

The model generated by the XGBoost algorithm, on the other hand, achieved higher classification accuracy than the *k*NN model across all three categories (WJ, RE, and NE), reaching 100% correct classifications during the training stage (Figure 5). However, in the testing stage, XGBoost exhibited slightly lower performance for the NE class, correctly classifying 24 out of 27 instances, and for the WJ class, with 53 out of 54 instances correctly identified. Similar to the results obtained with the *k*NN-based model, some misclassifications were observed between NE and RE samples, as well as between WJ and NE samples.

**Figure 5:**
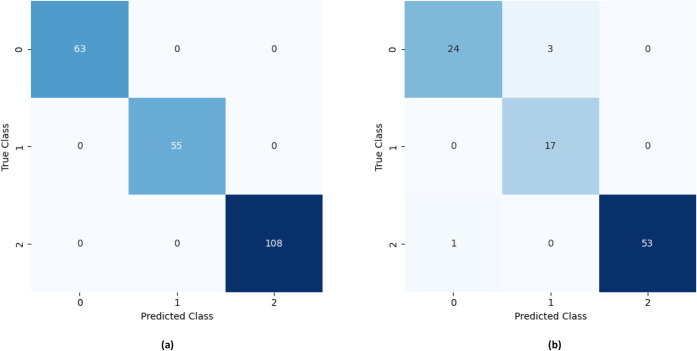
Confusion matrix for the classification model developed by the XGBoost algorithm, considering the (a) training and (b) testing stages. Code for classes: NE = 0; RE = 1; WJ = 2.

The higher training accuracy compared with the test set for both algorithms indicates a potential overfitting effect. Although such behavior is expected in datasets with limited sample sizes, the cross-validation results (*k*NN = 98.7%, XGBoost = 95.5%) together with the AUC curves (Figures S3 and S4) demonstrate that both models achieved good generalization performance. This outcome is attributable to the use of stratified metrics to address class imbalance among juice samples, which mitigated—albeit did not completely eliminate—the risk of overfitting.

Overall, both algorithms demonstrated good discriminative capability among apple juice categories, with *k*NN performing slightly better in generalization across all classes, particularly for WJ, while XGBoost showed greater stability in WJ and RE classification. These findings corroborate the results obtained from LDA analysis (Figure 3), which also revealed clear separation of WJ samples of other classes (NE and RE) and partial overlap between NE and RE beverages. Therefore, the supervised machine learning models not only confirmed the patterns previously identified through multivariate statistical analyses but also enhanced classification performance by capturing complex nonlinear relationships among image-derived chromatic variables.

To further assess the performance of the classification models generated by both algorithms, indices such as accuracy, precision, recall, *F1-score*, and overall accuracy were calculated (Table 1).

**Table 1:** Performance expressed in Percentage (%) of the Metrics for Classification Models Generated by the *k*NN and XGBoost Algorithms

Algorithm	Model	Class	Precision	Recall	*F1-Score*	Accuracy
*k*NN	Training	WJ	100.0	99.1	99.5	99.1
		RE	98.2	100.0	99.1	100.0
		NE	98.4	98.4	98.4	98.4
	Testing	WJ	100.0	96.3	98.1	96.3
		RE	89.5	100.0	94.4	100.0
		NE	92.6	92.6	92.6	92.6
XGBoost	Training	WJ	100.0	100.0	100.0	100.0
		RE	100.0	100.0	100.0	100.0
		NE	100.0	100.0	100.0	100.0
	Testing	WJ	100.0	98.1	99.1	98.1
		RE	85.0	100.0	91.9	100.0
		NE	100.0	98.1	99.1	88.9

The *k*NN-based model exhibited superior performance in classifying WJ, RE, and NE samples. During the testing stage, all performance metrics exceeded 89.5%. For the RE and WJ categories, *k*NN achieved accuracies of 100% and 99.5% in the training stage and 100% and 96.3% in testing stage, respectively (Table 1). Considering the overall classification accuracy (99.1% in training and 95.9% in testing), it produced a model with performance even exceeding, that reported by other authors for juice classification tasks, such as apple juice (49–83%),^11^ apple drinks (63%),^39^ lemon juice according to geographical origin (66.7%),^40^ adulterated lemon juice (88%),^44^ grape juice (57–89%),^45^ and fruit juice classification (93.3%).^46^

The XGBoost-based model achieved high accuracy for the classification of WJ (98.1%) and RE (100.0%). However, it produced a model with lower discriminative power for NE, achieving 88.9% accuracy in the testing stage (Table 1). Considering the overall accuracy (100.0% in training and 95.5% in testing), the XGBoost-based models for apple juice classification yielded estimates better than, or comparable to, those reported in studies involving grape berry ripeness (82.5–91.6%),^47^ coffee beans (86%),^48^ and Chinese dates (92.7–97.6%).^43^

These results demonstrate that both algorithms produced classification models capable of effectively discriminating among WJ, RE, and NE samples obtained through different technological processes. Notably, the XGBoost-based models achieved superior classification performance for WJ, which is particularly relevant given their higher commercial value and greater susceptibility to fraudulent or adulterated products. A limitation of this study was the relatively small dataset used for model training. Future work employing larger datasets, including apple juices produced through diverse technological processes and from different manufacturers, could yield classification models with improved generalization ability and practical applicability.

### 3.3 Predictive Modeling of Apple Juice

To evaluate whether image-derived data could predict apple juice concentration in different beverage categories (whole juice = 100%; reconstituted juices >50%; and nectars >20%), linear correlation analyses were performed using variables selected by VIF analysis (G, B, and H) and beverage concentrations (%), incorporating both calibration curve data (5–100% apple juice) and commercial apple juice samples (Table S2, Figure S2).

A positive correlation was observed between concentration (%) and variable G (*r* = 0.490, *p* < 0.001), while negative correlations were found for variables B (*r* = −0.729, *p* < 0.001) and H (*r* = −0.703, *p* < 0.001) (Figure 6). These results indicate that increasing apple juice concentration enhances the contribution of G, whereas reducing it increases the influence of B and H (hue) on the image data. This variation is most clearly observable in the calibration curve covering 5–100% apple juice (Figure S2).

**Figure 6:**
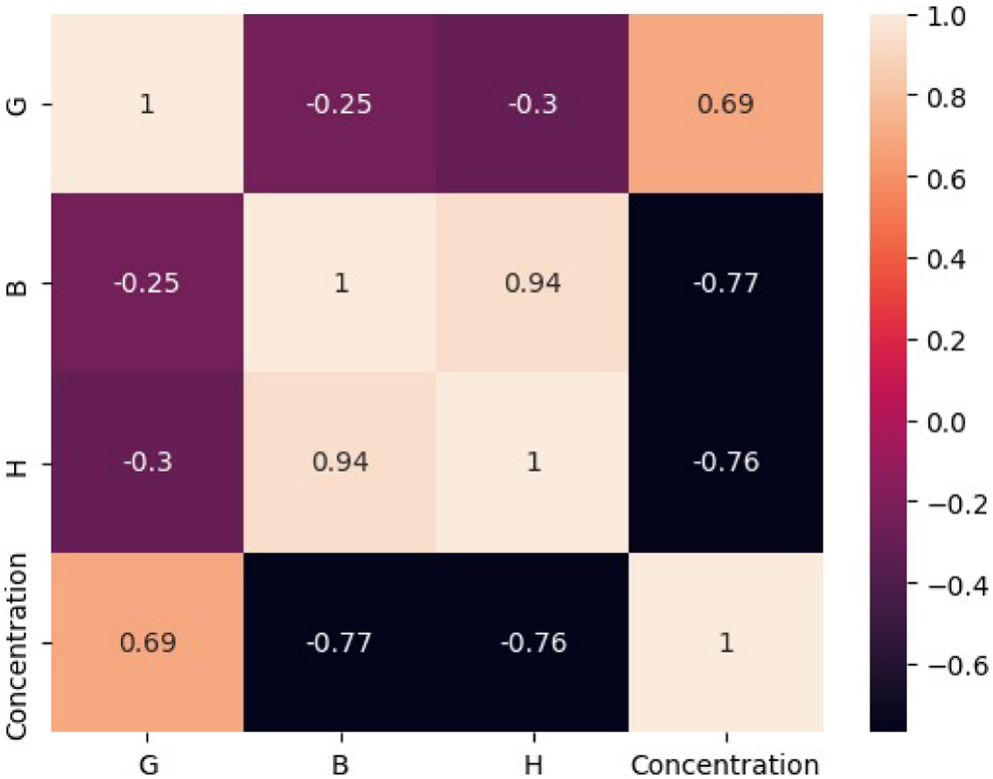
Linear correlation analysis between variables G, B, and H and apple juice concentration (%) added to apple beverages.

These findings highlight the potential for predictive modeling of apple juice concentration across its different forms (WJ, RE, and NE). Accordingly, two machine learning algorithms, XGBoost and CatBoost, were applied to the dataset, which included the calibration curve, commercial samples, and the VIF-selected variables (G, B, and H).

XGBoost-based models achieved *R* ^2^ values of 97.7% for training and 93.1% for testing, with prediction errors of 8.2% (RMSE) and 5.0% (MAE) (Figure 7A). CatBoost-based models demonstrated similar performance, with *R* ^2^ values of 96.2% and 93.5% for training and testing, and RMSE and MAE of 8.0% and 4.8%, respectively (Figure 7B). High cross-validation *R* ^2^ values (91.9% for XGBoost and 92.4% for CatBoost) indicate a low risk of overfitting, confirming the robustness and generalizability of both models.

**Figure 7:**
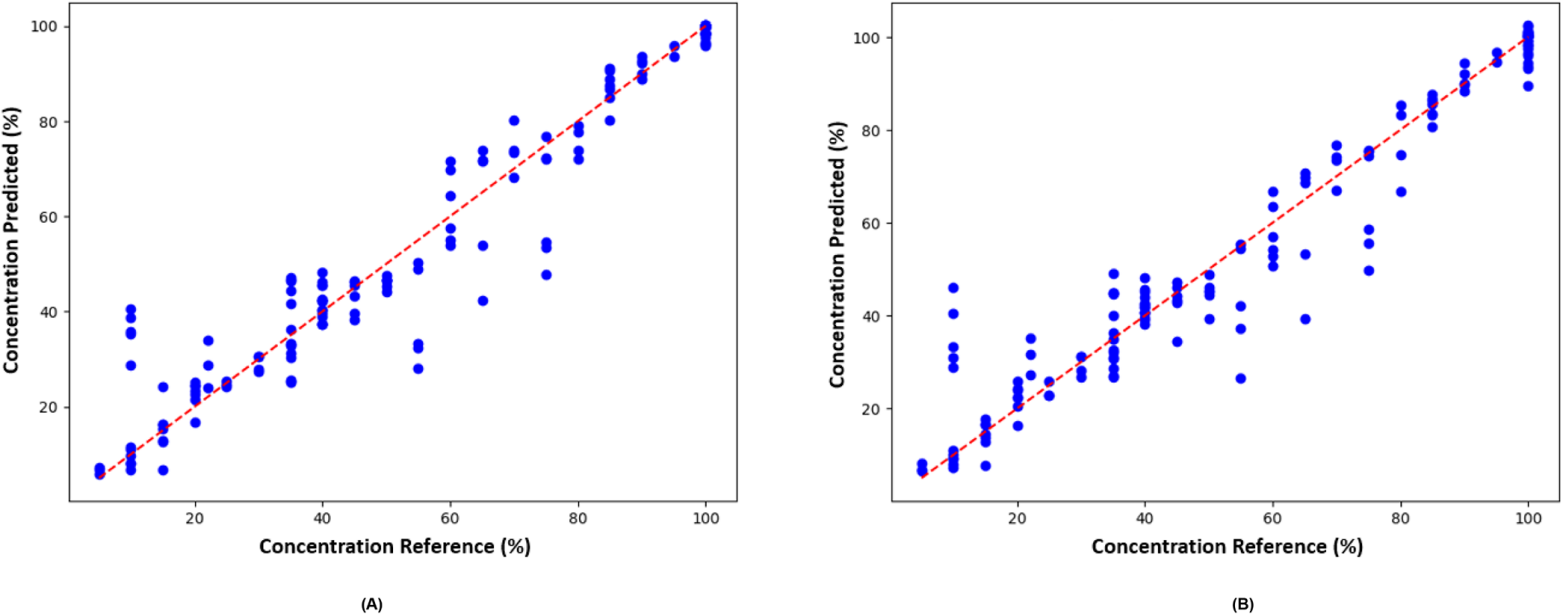
Plots of predicted values versus observed values (blue dots) for the prediction models generated by (A) XGBoost and (B) CatBoost algorithms.

Despite the high coefficients of determination, relatively high prediction errors were observed, which could compromise the accuracy and precision of the predictive models in regulatory or quality-control applications. This limitation could be addressed in future studies by using larger sample sets, typically available in industrial or regulatory contexts, along with independent cohorts and external validation, which would be essential to further substantiate the models’ generalizability.

A literature review revealed no prior applications of CatBoost or XGBoost algorithms for predicting fruit juice composition. However, these algorithms have been successfully applied to other food matrices using hyperspectral imaging. For example, Luo et al.^49^ predicted polyphenol content in teas (*R* ^2^ = 92.6–94.6%; RMSE = 4.3–5.0%), and Zou et al.^50^ predicted water content in potatoes (*R* ^2^ = 81.8–87.9%; RMSE = 5.0–8.0%). The models developed here for predict the apple juice content in beverages achieved comparable performance (*R* ^2^ = 93.1–93.5%; RMSE = 8.0–8.2%), demonstrating the excellent accuracy of both algorithms.

## 4 Conclusions

The analytical strategies developed in this study are rapid, cost-effective, and straightforward, combining data extracted from digital images of apple juice, captured using a smartphone, with machine learning algorithms. This integrated approach enabled accurate classification of apple-based beverages and reliable prediction of the apple juice content in whole juice, reconstituted juice, and nectar formulations.

The proposed classification and prediction models demonstrate strong potential for practical application. First, the methodology supports quality standardization in industrial production, promoting beverages with consistent compositional and organoleptic characteristics. Second, the classification strategy can contribute to fraud and adulteration prevention by allowing rapid identification of commercial apple juice categories, particularly whole juices. Third, the approaches are easily adaptable to both industrial and inspection laboratories, as they rely on low-cost instrumentation and are readily automated.

For broader implementation in regulatory and industrial environments, further studies should include modeling with larger datasets to enhance robustness and predictive accuracy. Future research should also explore the development of classification and prediction models for other fruit juices, particularly mixed beverages containing apple juice. Additionally, the robustness of image acquisition should be evaluated using different smartphone brands and under varying illumination conditions.

These findings highlight the potential of integrating digital image processing and machine learning as a sustainable, efficient, and accessible analytical tool for beverage authentication and quality control.

## Supplementary Material



## Notes

Information on apple juice samples declared on the labels by the manufacturers, the volumes of composite apple juice samples and distilled water used to construct the calibration curve in the 5–100% range, the ROIs of the apple juice samples and those used in the calibration curve adopted for the prediction modeling, as well as the AUC curves of the classification models (DOCX)
