# Supplementary material for: New Analytical Strategies for Quality Control and Classification of Apple Juices Using Digital Image Processing (DIP) Combined with Machine Learning (ML)
Source: ACS Omega. 2025 Dec 17;10(51):62828–38. doi: 10.1021/acsomega.5c08212 (PMC12756777; doi:10.1021/acsomega.5c08212)
Supplement: Supplementary file 1 [file ao5c08212_si_001.docx]

New Analytical Strategies for Quality Control and Classification of Apple Juices Using Digital Image Processing (DIP) Combined with Machine Learning (ML)

*Suelem Kaczala^a*^; Vanderlei Aparecido de Lima^b^; Maria Lurdes Felsner^a,c*^*

^a^ Department of Chemistry, State University of Midwestern at Paraná (UNICENTRO), Vila Carli, Zip Code 85040-080, Guarapuava city, Paraná, Brazil

^b^ Department of Chemistry, Federal University of Technology – Paraná (UTFPR), Zip Code 85503-390, Pato Branco city, Paraná, Brazil

^c^ Department of Chemistry, State University of Londrina (UEL), Zip Code 86057-970, Londrina city, Paraná, Brazil

Suelem Kaczala. E-mail: suelemkaczala@gmail.com

Vanderlei Aparecido de Lima. E-mail: [valima@utfpr.edu.br](mailto:valima@utfpr.edu.br)

Maria Lurdes Felsner. E-mail: [felsner@unicentro.br](mailto:felsner@unicentro.br); [felsner@uel.br](mailto:felsner@uel.br)

*Corresponding author at: Department of Chemistry. State University of Midwest in Paraná; Zip Code 85040-080, Guarapuava city, Paraná, Brazil. Phone: ++55 46 9935-8321; E-mail address: [suelemkaczala@gmail.com](mailto:suelemkaczala@gmail.com) (S. Kaczala); [felsner@unicentro.br](mailto:felsner@unicentro.br) (M. L. Felsner).

**Table S1.** Manufacturer-declared information on the labels of the apple juice samples.

| **Juice Type** | **Sample Coding** | **Processing*** | **Manufacturer** | **Composition Declared on the Label by the Manufacturer** |
| --- | --- | --- | --- | --- |
| Whole (WJ)  (n = 9) | WJ2  WJ6  WJ7 | WC  WC  C | B  F  G | Whole apple juice and ascorbic acid (INS 300) |
|  | WJ3  WJ9 | C | C  I | Whole apple juice |
|  | WJ8 | WC | H | Whole and reconstituted apple juice |
|  | WJ1 | WC | A | Concentrated apple juice, ascorbic acid (INS 300) and citric acid (INS 330) |
|  | WJ4 | WC | D | Whole apple juice, ascorbic acid (INS 300) and sulphur dioxide (INS 220) |
|  | WJ5 | WC | E | Whole apple juice, ascorbic acid (INS 300) and potassium metabisulfite (INS 223) |
| Reconstituted (RE)  (n = 4) | RE1  RE3 | C  C | J  A | Concentrated apple juice and water |
|  | RE2 | WC | M | Concentrated apple juice, water and citric acid (INS 330) |
|  | RE4 | WC | Q | Concentrated apple juice, water, citric acid (INS 330) and ascorbic acid (INS 300) |
| Nectars (NE)  (n = 5) | NE1 | C | K | Concentrated apple juice, water, sugar, ascorbic acid (INS 300), malic acid (INS 296), sodium citrate (INS 331), caramel dye, acesulfame potassium (INS 950) and sucralose  Concentration of apple juice in formulated beverage = 35 % |
|  | NE2 | C | L | Concentrated apple juice, water, ascorbic acid (INS 300), malic acid (INS 296), citric acid (INS 330),  acesulfame potassium (INS 950) and sucralose  Concentration of apple juice in formulated beverage = 50 % |
|  | NE3 | C | N | Concentrated apple juice, lemon juice, water, sugar, citric acid (INS 330), vitamins, guar gum (INS 412) and carotene and anthocyanin dyes (INS 163)  Concentration of apple juice in formulated beverage = 10 % |
|  | NE4 | WC | O | Concentrated apple juice, water, sugar, calcium, vitamins, citric acid (INS 330) and xanthan gum (INS 415)  Concentration of apple juice in formulated beverage = 22 % |
|  | NE5 | C | P | Concentrated apple juice, water, sugar, citric acid (INS 330), ascorbic acid (INS 300), flavoring, acesulfame potassium (INS 950), sucralose, xanthan gum (INS 415) and anti-foaming agent  Concentration of apple juice in formulated beverage = 40 % |

*** WC = non clarified and filtered juice; C = clarified and filtered juice

**Table S2**. Volumes of the composite apple juice sample and distilled water used to prepare the calibration curve (5–100% apple juice).

| **Components** | **Concentration of Standard Solutions (%)** | | | | | | | | | | | | | | | | | | | |
| --- | --- | --- | --- | --- | --- | --- | --- | --- | --- | --- | --- | --- | --- | --- | --- | --- | --- | --- | --- | --- |
|  | **100** | **95** | **90** | **85** | **80** | **75** | **70** | **65** | **60** | **55** | **50** | **45** | **40** | **35** | **30** | **25** | **20** | **15** | **10** | **5** |
| Apple juice composite sample (mL) | 10 | 9,5 | 9,0 | 8,5 | 8,0 | 7,5 | 7,0 | 6,5 | 6,0 | 5,5 | 5,0 | 4,5 | 4,0 | 3,5 | 3,0 | 2,5 | 2,0 | 1,5 | 1,0 | 0,5 |
| Water  (mL) | 0 | 0,5 | 1,0 | 1,5 | 2,0 | 2,5 | 3,0 | 3,5 | 4,0 | 4,5 | 5,0 | 5,5 | 6,0 | 6,5 | 7,0 | 7,5 | 8,0 | 8,5 | 9,0 | 9,5 |


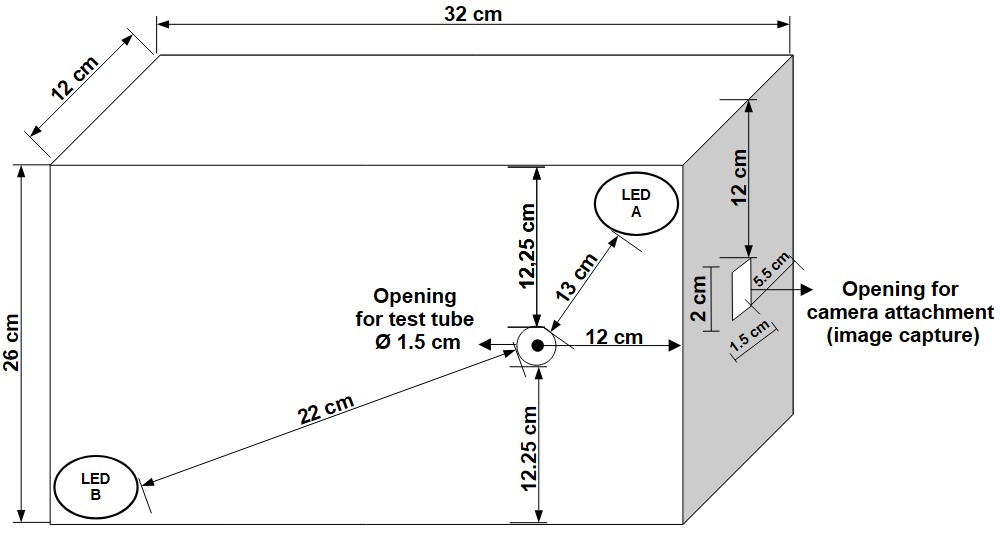


**Figure S1.** Schematic diagram of the top view of the cardboard image acquisition chamber showing all dimensions and openings for smartphone camera attachment and test tube placement.

**(b)**

**
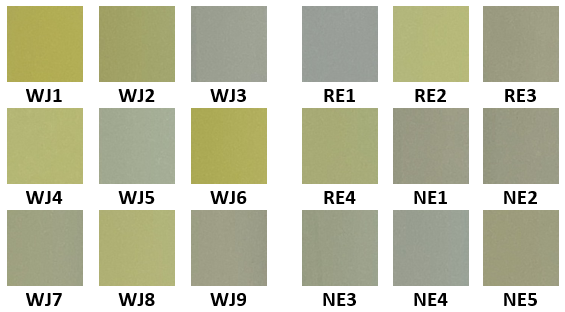
**

**(a)**

**(c)**


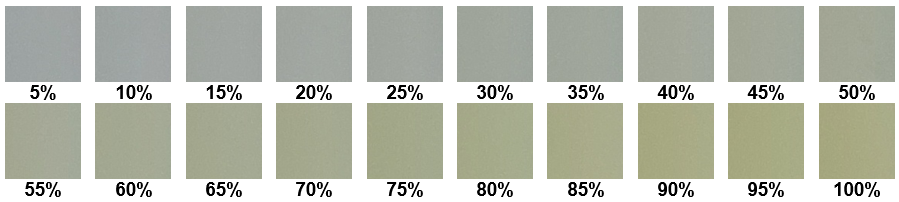


**(c)**

**Figure S2.** (a) Illustration of 100 × 100 pixel ROIs extracted from images of apple juice samples; (b) ROIs of whole juice (WJ), reconstituted juice (RE), and nectar (NE) samples used in the classification models; (c) ROIs corresponding to calibration curve points ranging from 5% to 100% apple juice, used in the prediction model


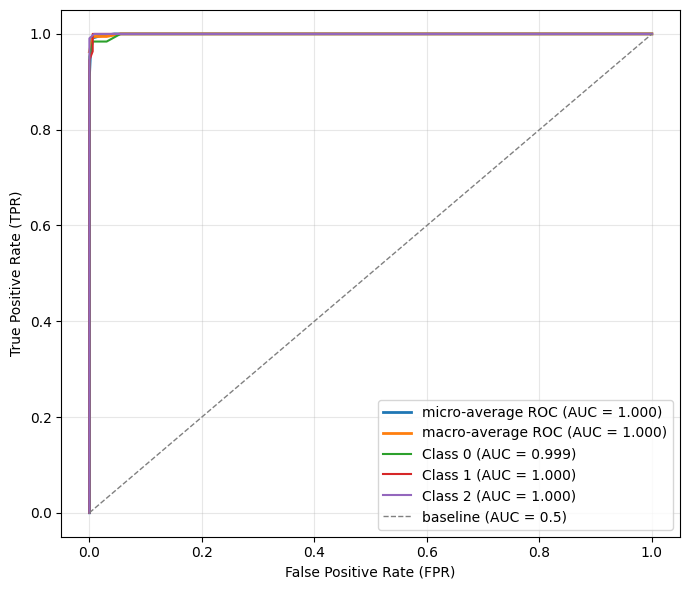

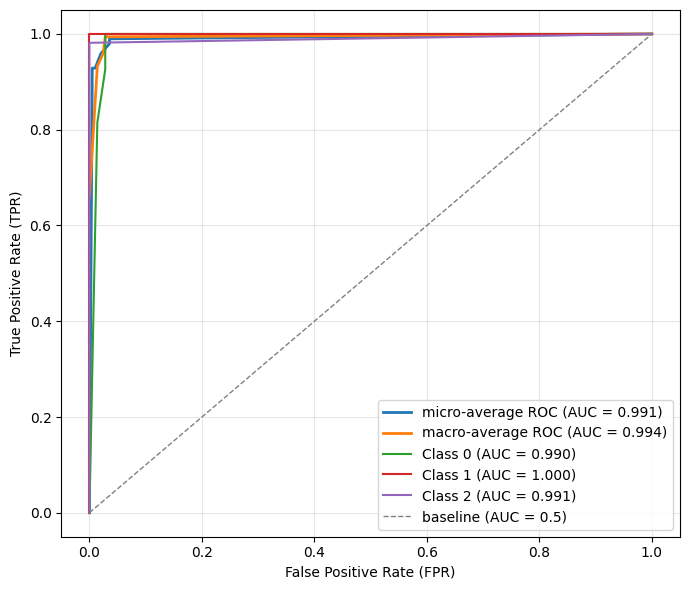


1. **(b)**

**Figure S3.** AUC curves of the classification models generated by the *k*NN algorithm during the (a) training and (b) testing stages. Codification for categories of apple juice: NE = 0; RE = 1; WJ = 2


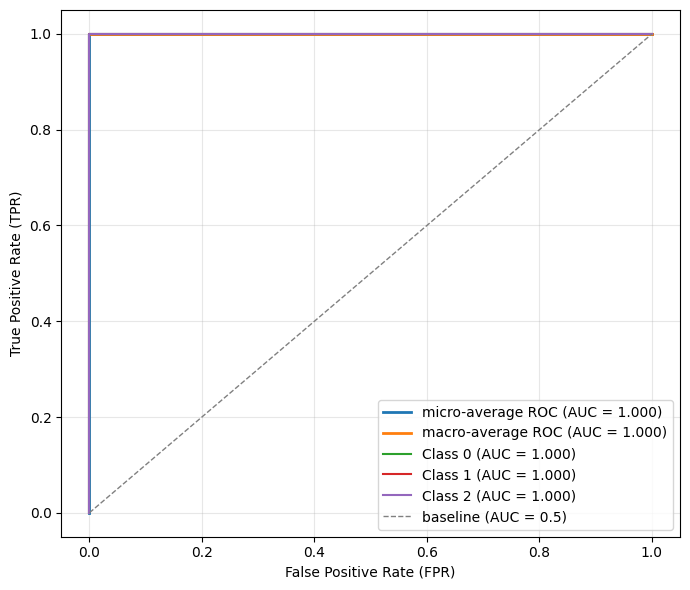

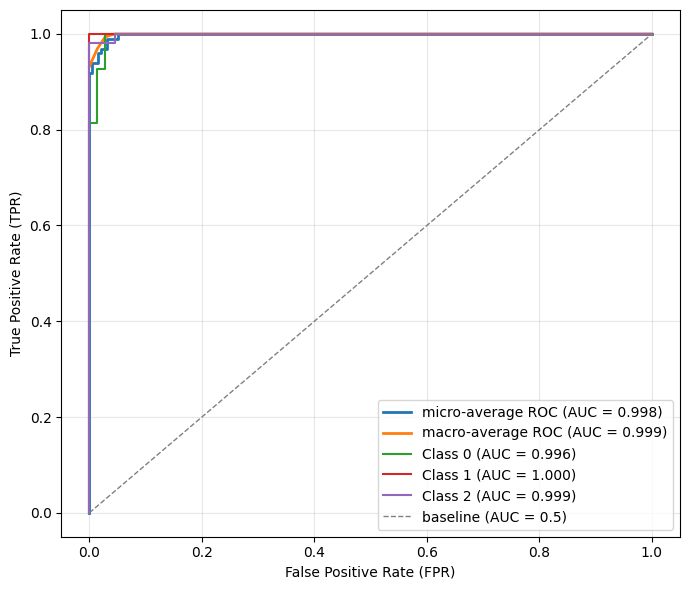


1. **(b)**

**Figure S4.** AUC curves of the classification models generated by the XGBoost algorithm during the (a) training and (b) testing stages. Codification for categories of apple juice: NE = 0; RE = 1; WJ = 2
